# Comparative Genomics and Draft Genome Assembly of the Elite Tunisian Date Palm Cultivar Deglet Nour: Insights into the Genetic Variations Linked to Fruit Ripening and Quality Traits

**DOI:** 10.3390/ijms26146844

**Published:** 2025-07-16

**Authors:** Rahma Zarkouna, Afifa Hachef, Carmine Fruggiero, Gaetano Aufiero, Davide D’Angelo, Hedia Bourguiba, Maha Mezghani-Khemakhem, Nunzio D’Agostino, Salwa Zehdi-Azouzi

**Affiliations:** 1 Laboratory of Molecular Genetics, Immunology and Biotechnology (LR99ES12), Faculty of Sciences of Tunis, University of Tunis El Manar, El Manar I, Tunis 2092, Tunisia; rahma.zarkouna@etudiant-fst.utm.tn (R.Z.); afifa.hachef@fst.utm.tn (A.H.); hedia.bourguiba@fst.utm.tn (H.B.); maha.mezghani@fst.utm.tn (M.M.-K.); salwa.zehdi@fst.utm.tn (S.Z.-A.); 2 Department of Agricultural Sciences, University of Naples Federico II, Piazza Carlo di Borbone 1, 80055 Portici, Italy; carmine.fruggiero@unina.it (C.F.); gaetano.aufiero@unina.it (G.A.); davide.dangelo@unina.it (D.D.)

**Keywords:** *Phoenix dactylifera*, genome resequencing, nucleotide variations, fruit ripening, fruit quality

## Abstract

The date palm (*Phoenix dactylifera* L.) is a key crop in the arid regions of North Africa and the Middle East, with substantial socioeconomic value. Although multiple genome assemblies have been generated using next-generation sequencing (NGS) technologies, they primarily focus on Middle Eastern cultivars, leaving North African varieties unrepresented. This study aims to address this gap by sequencing and assembling the first genome of a North African date palm using Illumina sequencing technology. We present a draft genome assembly of the elite Tunisian variety Deglet Nour. By comparing it with the Barhee BC4 reference genome, we identify key genetic variants, including single nucleotide polymorphisms (SNPs) and insertions/deletions (INDELs), potentially associated with ripening processes and fruit quality. This work expands the genomic resources for date palm research, particularly for North African cultivars, and provides new insights into the nucleotide-level variability of the genes linked to key agronomic traits.

## 1. Introduction

Over the past two decades, rapid advances in plant genome sequencing have significantly increased both the quantity and the quality of publicly available genomic resources. This growth has opened up new avenues for exploring genome biology and the evolutionary dynamics of land plants. The growing body of genomic data from diverse plant taxa, including major crops, has deepened our understanding of plant development, adaptation, and the genetic basis of natural and artificial selection.

Genome sequencing allows for the identification and functional analysis of genes associated with key plant traits, such as disease resistance, stress tolerance, and crop yield and quality. Numerous in silico studies have been conducted to detect genetic variants associated with selection, genetic diversity, and variation in specific genes of interest [1,2,3,4]. This, in turn, facilitates targeted crop improvement, enhancing adaptation research and trait selection. These breakthroughs are paving the way for innovations in sustainable agriculture, as genomic insights are being used to develop crops that are more resilient against environmental stresses, ultimately supporting global food security [5,6].

The date palm (*Phoenix dactylifera* L.) is a dioecious plant (2n = 36), belonging to the *Arecaceae* family and the *Phoenix* genus, with significant socioeconomic importance in the Middle East and North Africa. Numerous studies have focused on various aspects of *P. dactylifera*, including the genetic diversity [7,8] and sex differentiation [9]. These studies showed that the date palm displays high genetic diversity and that the genetic variation is geographically structured in two pools, Eastern and Western. The Eastern pool includes accessions from Asia and Djibouti, whilst the Western pool consists of accessions from Africa. The significant differences between the Eastern and Western accessions suggest that each pool likely has its own distinct autochthonous origin.

The first genome sequencing of the date palm by Al-Dous et al. (2011) [10], based on the Khalas variety from Qatar, laid a foundational framework for investigating key agronomic traits such as fruit development, sex differentiation, and stress tolerance. This work was further refined by Al-Mssallem et al. (2013) [11], who improved the genome assembly and expanded the gene annotations. The most comprehensive reference genome to date was produced by Hazzouri et al. (2019) [12], using long-read sequencing to assemble the genome of the male Barhee BC4 cultivar. This high-quality assembly enabled genome-wide association studies (GWAS), leading to the identification of loci linked to fruit color, sugar composition, and sex determination—traits central to fruit quality and domestication. Despite these advances, genomic resources remain heavily biased toward cultivars from the Arabian Gulf region [12,13]. North African cultivars, by contrast, have been largely overlooked, despite their broad cultivation and unique agronomic potential. Few studies have explored the genomic architecture or adaptive traits of Deglet Nour, the most iconic and commercially significant variety in Tunisia and Algeria, renowned for its distinctive phenotypic characteristics. This underrepresentation has created a critical knowledge gap, limiting region-specific breeding efforts and hindering our understanding of traits important for adaptation to North African agro-ecological conditions.

**Figure 1:**
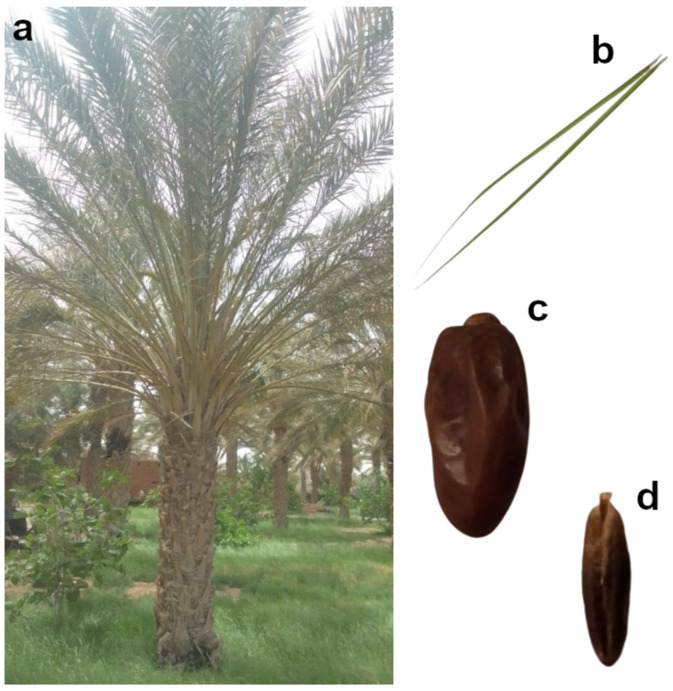
Morphological characteristics of the elite Tunisian date palm cultivar Deglet Nour. (**a**) Mature female palm; (**b**) leaf; (**c**) ripe dark brown fruit; and (**d**) date seed. Photos by Dr. Afifa Hachef and Rahma Zarkouna.

In Tunisia, the date palm plays a central role in the agricultural sector and holds profound economic, cultural, and religious significance. With around 250 varieties cataloged by Rhouma (1994, 2005) [14,15], it is among the most studied crops in the region, particularly in terms of efforts to improve cultivation and ensure sustainability. Among these, Deglet Nour stands out as the elite cultivar, widely grown and considered the most suitable candidate for genetic research on North African date palms (Figure 1).

Complementing nuclear genome efforts, organellar genome studies have also advanced our understanding of varietal differentiation and evolutionary relationships. Early work focused on general plastid [16] and mitochondrial [17] genomes, while more recent studies [18] have provided complete chloroplast and mitochondrial genome sequences for several Tunisian cultivars, including Deglet Nour. These data highlight the genetic distinctiveness of North African varieties and underscore the need for comprehensive nuclear genome assemblies to fully capture their genomic landscape.

To address this gap, we present the first draft nuclear genome assembly of Deglet Nour, constructed using Illumina short-read sequencing. This resource represents a significant step toward diversifying the date palm genomic landscape and establishing a reference for North African germplasm. Comparative analysis with the elite Gulf region cultivar Barhee BC4—a male genotype derived from a fourth-generation backcross with a female Barhee [12]—enables the identification of key genomic variants, including single nucleotide polymorphisms (SNPs) and insertions/deletions (INDELs). Several of these variants are potentially linked to important traits such as fruit texture, sugar metabolism, and ripening time.

By generating this reference genome, our study provides a foundational resource for genomics-assisted breeding, conservation, and improvement of North African date palms. It addresses a critical gap in date palm genomics and supports the development of strategies tailored to the unique agro-ecological conditions of North Africa. More broadly, it contributes to regional and global efforts aimed at enhancing fruit quality, stress resilience, and climate adaptation in this important crop.

## 2. Results

### 2.1. Draft Genome Assembly and Annotation

Illumina sequencing using 2 × 151 bp paired-end short-read insert libraries yielded approximately 172.7 million reads for both the R1 and R2 datasets. Table 1 summarizes the sequencing data quality and processing results. After processing, both FASTQ files retained 157.9 million paired reads. However, the number of reads where only one mate from a pair survived differed substantially between the two files. R1 had 9,766,454 single-end reads remaining, while R2 had only 2,324,394. This indicates that R1 experienced greater read pair disruption or quality loss during the processing steps.

Considering only the high-quality paired-end reads, we estimated the genome coverage based on two reference genome size assumptions. Using a genome size of 772 Mb [12], we obtained a target coverage of approximately 62×. Alternatively, using the 980 Mb genome size estimated by Fulgent microdensitometry from the Plant DNA C-value database (https://cvalues.science.kew.org/ accessed on 14 July 2025) [19], the coverage was approximately 49×.

The assembled genome has a total size of 431 Mb (Table 2; Figure S1), representing approximately 56% of the estimated 772 Mb genome size of the Barhee BC4 cultivar. Alternatively, using the 980 Mb estimate from the Plant DNA C-value database, the assembly covers about 44% of the genome. The assembly comprises 16,167 contigs, with an N50 of 12,215 Kb and an N90 of 36,742 bp. These metrics together provide a more complete picture of the assembly’s continuity, capturing both the largest and the smaller contigs. The GC content is 38.62% (Figure S2). Table 2 presents a comparison of the Deglet Nour date palm genome assembly statistics with those of previously published assemblies available in GenBank.

Furthermore, an annotation lifted from the Barhee BC4 reference allowed the prediction of 29,856 genes and extensive structural gene information. The estimated heterozygosity rate is 0.6%, with a total of 1,062,681 SNPs and 114,307 INDELs (Table 3).

### 2.2. Genome Assembly Assessment

The genome statistics for the date palm Deglet Nour draft genome provide valuable insights into the quality and completeness of the assembly. The overall genome fraction stands at 47.35%, representing the proportion of the genome captured in the assembly.

One noteworthy metric is the duplication ratio of 1.026, indicating a well-balanced assembly with neither excessive duplication nor significant underrepresentation of genomic regions—values close to 1 are generally ideal. The number of ambiguous or unresolved bases, represented by the N’s per 100 kbp, is 8585, reflecting relatively complete assemblies but highlighting regions where sequence data remains uncertain or missing, which may require further sequencing or refinement. Remapping of the 157.9 million paired reads used in the assembly shows an overall alignment rate of 87.09%, demonstrating a high level of read alignment. The mean per-base sequencing depth across the genome assembly is 94.26×.

**Figure 2:**
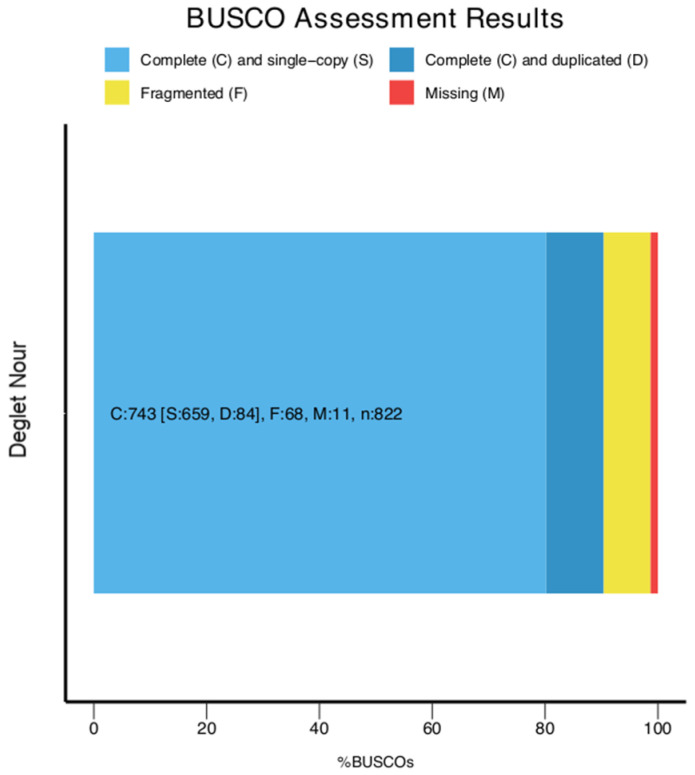
BUSCO-generated bar chart depicting the gene completeness based on the viridiplantae_odb12 dataset. The chart displays the proportion of complete, fragmented, and missing BUSCO genes.

BUSCO analysis was performed to assess the completeness of the final genome assembly using a set of 822 conserved orthologs (i.e., viridiplantae_odb12 dataset). The results indicate a high level of completeness, with 90.4% of the BUSCO genes detected as complete. Of these, 80.2% are present as single-copy genes, while 10.2% are duplicated. Fragmented BUSCOs account for 8.3%, and only 1.3% of the BUSCOs are missing from the assembly. Notably, 45 of the complete BUSCO genes contain internal stop codons, suggesting potential gene model issues. These results demonstrate that the final assembly captures the vast majority of expected conserved orthologs (Figure 2). Synteny analysis revealed that 47% of genes are conserved in the same relative order and orientation across all the corresponding chromosomes. This moderate level of collinearity reflects either incomplete or fragmented scaffolds that artificially disrupt syntenic blocks or genuine structural rearrangements. Despite these disruptions, each of the 18 largest scaffolds from Deglet Nour aligns uniquely and corresponds to a single reference chromosome from Barhee BC4, establishing a clear one-to-one syntenic relationship.

**Figure 3:**
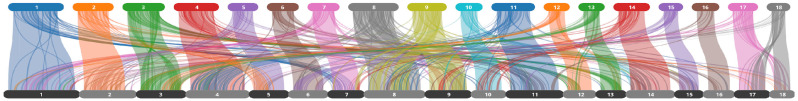
MCScanX pairwise alignment between the first 18 scaffolds of the Deglet Nour genome (top) and the first 18 chromosomes of the Barhee BC4 reference genome (bottom). Genomic sequences are numerically labeled. Collinear gene blocks—defined as regions containing at least five homologous genes with a maximum intergenic gap of 20 genes—are visualized as ribbons connecting the two genomes. The ribbon thickness is proportional to the number of genes in each block, while the ribbon color indicates the originating Deglet Nour scaffold.

However, the Deglet Nour scaffolds tend to be shorter and exhibit local gene-level rearrangements (Figure 3), suggesting some degree of genomic structural variation between the two cultivars. These local rearrangements are exemplified by four inversions identified between scaffold 1 and reference chromosome 1, each spanning approximately 8 to 10 genes (Figure S3). The inversions are located on the reference genome at approximately 2.2 million, 11.7 million, 24.4 million, and 40.6 million base pairs, respectively.

### 2.3. Transposable Elements

The combined analysis using RepeatMasker and DANTE identified a total of 62,891 transposable elements (TEs) in the Deglet Nour genome, accounting for approximately 48.44% of its total length. Both Class I (retrotransposons) and Class II (DNA transposons) were detected (Table S1). The Class I elements included long interspersed nuclear elements (LINEs; N. = 11,818), long terminal repeats (LTRs; N. = 44,803), and short interspersed nuclear elements (SINEs; 2530), with the LTRs being the most abundant. Among the LTRs, the Gypsy (N. = 18,911) and Copia (N. = 25,892) superfamilies dominated. The Class II elements were divided into two subclasses: Subclass 1, comprising terminal inverted repeats (TIRs; N. = 3,193), and Subclass 2, consisting of Helitrons. Within the TIR order, the hAT superfamily was the most prevalent, with 2001 elements identified.

### 2.4. Annotation of Variants by SnpEff

**Figure 4:**
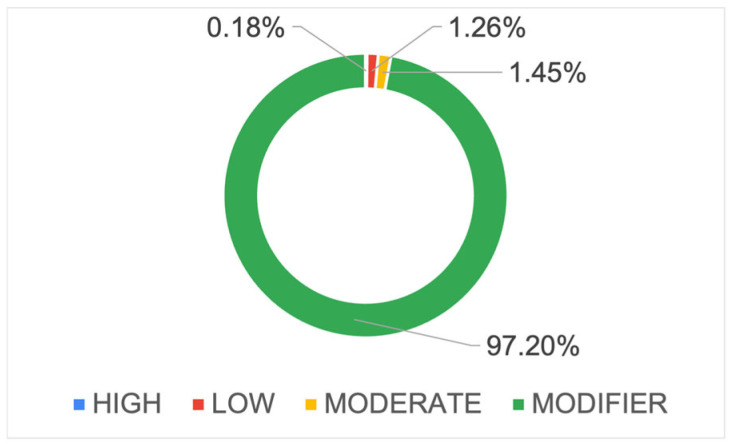
Pie chart showing the percentage distribution of the variant impact categories: HIGH, LOW, MODERATE, and MODIFIER.

The identified variants were annotated and classified into four impact categories using SnpEff: MODIFIER (~97.1%), MODERATE (~1.4%), LOW (~1.2%), and HIGH (~0.18%) (Figure 4; Table S2).

**Figure 5:**
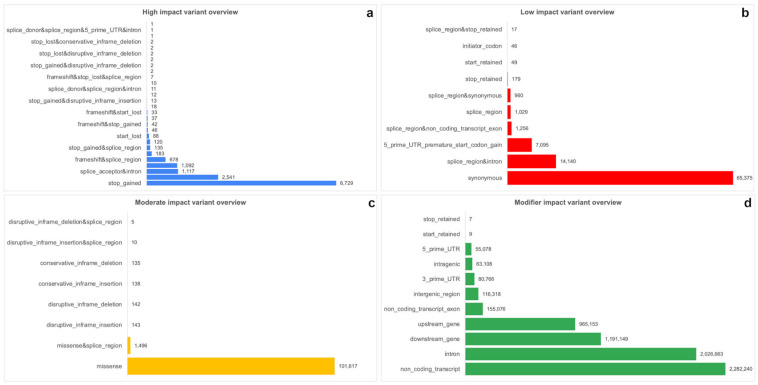
(**a**–**d**) Bar charts illustrating the detailed frequency and overview of the variants within each category: (**a**) HIGH, (**b**) LOW, (**c**) MODERATE, and (**d**) MODIFIER impact variants.

Each impact category is associated with specific types of functional effects and distributed across various genomic regions (Figure 5a–d). Within the LOW impact category, the most prevalent variant type was synonymous variants. In the MODIFIER category, non-coding transcript variants were dominant. The MODERATE category was primarily composed of missense variants, while the HIGH impact category was characterized mainly by stop-gained variants, representing the most severe functional consequences.

Furthermore, numerous genetic variations were observed in genes associated with the ripening process and quality traits of date palm fruits (Table S3). Variants were identified in genes involved in sucrose metabolism, which plays a crucial role in determining the fruit sweetness and energy balance. Additionally, variations were detected in genes linked to fruit shape, size, and weight, suggesting a potential genetic basis for the morphological diversity among date palm cultivars. Genes related to fruit firmness also exhibited notable variation, which may influence the textural properties during ripening and post-harvest handling (Table S4).

## 3. Discussion

### 3.1. Genome Assembly and Basic Features

This study presents the draft genome assembly of the Tunisian date palm cultivar Deglet Nour, with an assembled genome size of 431 Mb.

The sequencing effort resulted in a high mean per-base depth of 94.26× across the assembled genome. This depth reflects the combined influence of the sequencing yield, read quality, and mapping efficiency, with 87.09% of the 157.9 million high-quality paired-end reads successfully aligning with the assembly—highlighting its overall integrity. Coverage above 30× is generally considered sufficient for accurate base calling, structural variant detection, and contig assembly in Illumina-based projects. Thus, the depth achieved here exceeds the typical thresholds and supports high-confidence applications such as de novo assembly, gene annotation, and variant discovery.

The assembly shows strong continuity, as indicated by the N50 value (12.215 Kb), reflecting substantial large contigs. However, the N90 metric (36,742 bp) reveals the presence of numerous smaller contigs, providing a more balanced perspective on the overall fragmentation and quality of the assembly. Evaluating both measures together offers a fuller understanding of the contig size distribution and assembly integrity.

The scaffolding and annotation were performed using the male Barhee BC4 genome (772 Mb) as a reference [12]. The GC content of the Deglet Nour genome is 38.62%, closely aligning with both the Khalas variety genome published in 2011 (38.5%) [10] and the Barhee reference genome [12]. The estimated heterozygosity rate is 0.6%, which is comparable to the 0.46% reported for the Khalas genome [10]. Although relatively low for a dioecious and outcrossing species, this heterozygosity level likely reflects the specific genetic background of the Deglet Nour cultivar and the unique population structure of date palms from the Tozeur region in southern Tunisia. The sample used in this study is an offshoot from a female Deglet Nour palm that has been cultivated in the Tozeur region since the 1980s. Previous studies using SSR markers have shown that the date palm populations in the Tozeur oasis exhibit a deficiency in heterozygosity [7]. This reduced heterozygosity may indicate limited genetic diversity within the local population, possibly resulting from long-term clonal propagation practices and geographic isolation, which together shape the genomic landscape of Deglet Nour. Importantly, these genetic features could underlie some of the cultivar’s unique phenotypic traits and its adaptation to the harsh environmental conditions characteristic of southern Tunisia’s oases. Such a pattern has important implications for conservation and breeding strategies, as low genetic variation can reduce the adaptive potential and resilience against environmental stresses or emerging pests and diseases. Therefore, introducing new genetic diversity while preserving the unique genomic identity of Deglet Nour will be essential to bolster its adaptive capacity and ensure sustainable cultivation in the region.

### 3.2. Transposable Element Composition

In the Deglet Nour genome, the transposable elements (TEs) are predominantly long terminal repeat (LTR) retrotransposons of Class I, comprising approximately 35% of the total transposable elements in the genome. The Copia and Gypsy superfamilies are the most abundant among these LTR elements. Within the Class II DNA transposon, terminal inverted repeats (TIRs) are the most abundant. This TE composition mirrors findings from the first published date palm genome by Al-Dous et al. (2011) [10], which also reported Copia and Gypsy as the dominant LTR families and identified the CACTA family as the most prevalent DNA transposon. Similarly, the Barhee BC4 reference genome shows LTR elements as the major TE class [12].

The dominance of LTR retrotransposons, particularly Copia and Gypsy, likely contributes to the genetic diversity and adaptation by affecting the gene regulation and genome plasticity [20,21]. The overall TE content in Deglet Nour (48.4%) is consistent with other palms, such as oil palm (*Elaeis guineensis*; ~57%) [22,23], though it is lower than in coconut (*Cocos nucifera*; ~72.8%) [24]. These comparisons position the Deglet Nour genome within the expected range of repetitive content for *Arecaceae* species.

### 3.3. Genome-Wide Variant Detection

A total of 1,062,681 SNPs were detected in the Deglet Nour genome, along with 63,274 insertions and 51,033 deletions. Through the annotation of these variants and a review of the relevant literature, we identified several genes of interest carrying variants associated with key agronomic traits in date palms. Most of the identified variants within the coding regions were missense mutations with moderate impact, while a smaller proportion consisted of high-impact variants, such as frameshift mutations and stop-gained or -lost changes, that may have significant effects on protein function.

#### 3.3.1. Variants in Sugar Metabolism Genes

Several genes involved in sugar metabolism were found to carry missense variants with potential functional significance. The neutral/alkaline invertase 3 gene (LOC103706133) exhibited three missense variants of moderate impact, while beta-fructofuranosidase (CWINV1) (LOC103698975) harbored two similar variants. In addition, beta-fructofuranosidase, insoluble isoenzyme 3-like (LOC103713368), beta-fructofuranosidase 1 (LOC103705165), and sucrose synthase 1 (LOC103702434) each contained multiple missense variants with moderate impact.

These genes have previously been studied for their roles in sugar metabolism during fruit development [11,25], and significant GWAS signals have been linked to invertase-related loci [12]. Notably, neutral/alkaline invertase 3, which catalyzes the hydrolysis of sucrose under a neutral to alkaline pH, is upregulated during the late stages of date fruit maturation, coinciding with rapid sugar accumulation [10,24]. Similarly, the expression of CWINV1 increases during the final maturation phase, reflecting a shift in the soluble sugar content, and multiple gene copies and sequence variants have been identified, underscoring its importance in terms of the sugar composition [26].

Sucrose synthase 1, which catalyzes the reversible conversion of sucrose and UDP to UDP-glucose and -fructose, plays a key role in providing substrates for energy metabolism and cell wall biosynthesis during fruit expansion [27]. Differential gene expression analyses during various stages of fruit development [11] and functional pathway studies [28] highlight the contributions of these genes to the sucrose and starch metabolism network.

Together, these findings underscore the importance of neutral/alkaline invertase 3, beta-fructofuranosidase isoforms, and sucrose synthase 1 as key regulators of sugar content and sweetness in Deglet Nour.

#### 3.3.2. Variants in Fruit Shape, Size, and Weight Genes

We identified several genes with known roles in fruit morphology that exhibited notable variants in the Deglet Nour genome. The OVATE family protein (LOC103713458), an ortholog of a major shape-regulating gene first characterized in tomato (AAN17752.1), contained multiple variants within its coding region. The OVATE family proteins are key regulators of fruit shape, modulating the cell division patterns and organ morphogenesis. In tomato, OVATE interacts with other loci such as fw2.2 to produce a pear-shaped fruit morphology [29,30,31]. This gene has also been the focus of multiple QTL mapping studies [32,33], with homologs and associated QTLs identified in other fruit crops, such as papaya [34].

Similarly, we identified two orthologs of the well-characterized Cell Number Regulator gene fw2.2—LOC120103770 and LOC103712501—both of which contained coding sequence variants, including a high-impact stop-gained variant in LOC120103770. The fw2.2 locus is a major determinant of fruit size, explaining up to 30% of the size variation between wild and cultivated tomato lines by negatively regulating cell division during early fruit development [35]. The phenotypic effects of fw2.2 are largely attributed to regulatory mutations that alter the timing of gene expression, influencing mitotic activity during early fruit development [36,37]. In addition, fw2.2 has been shown to affect intercellular signaling by modulating the plasmodesmata permeability through callose deposition, thereby influencing fruit growth at the tissue level. Its function appears conserved among other Cell Number Regulator family members in various plant species [38], including papaya [34].

Furthermore, we identified additional fruit weight-associated genes, including Trichome birefringence-like 12 (LOC103723990) and Glutamate receptor 3 (LOC120110542), orthologs of the *Carica papaya* proteins LOC110818944 and LOC110821828, respectively. Several variants were detected in both genes, notably a high-impact frameshift mutation in the Glutamate receptor 3 gene. Trichome birefringence-like 12 is involved in cell wall modification processes essential for cell expansion and fruit growth, while Glutamate receptor 3 may influence fruit size through cellular signaling pathways, though its precise mechanisms remain to be fully elucidated. These genes have previously been mapped to quantitative trait loci (QTL) regions associated with fruit size and weight variation in papaya [39].

#### 3.3.3. Variants in Fruit Firmness Genes

Fruit firmness, a key determinant of date quality, was associated with several genes in the Deglet Nour genome that carried potentially impactful variants. Three orthologs of the Expansin (EXP1) gene—LOC103706420, LOC103716383, and LOC103719608—each harbored missense variants of moderate impact. Expansins are cell-wall-loosening proteins that facilitate fruit softening by disrupting the noncovalent bonds between cellulose and hemicellulose, enhancing the accessibility of wall components to degradation enzymes [40,41]. In tomato, LeExp1 is highly expressed during ripening, and CRISPR/Cas9 studies have shown that simultaneous knockout of SlExp1 and SlCel2 increases firmness by limiting pectin and xyloglucan degradation [42]. Similar expression dynamics have been reported for multiple FaEXP genes in strawberry [41].

Two orthologs of the MADS-box transcription factor RIN—LOC103712799 and LOC103702755—were also identified, with LOC103712799 carrying a high-impact frameshift variant. In tomato, RIN is a central regulator of ripening that controls downstream genes linked to ethylene biosynthesis, texture, and flavor. Loss-of-function mutations (e.g., rin) result in impaired ripening, and RIN has been shown to interact with CNR-SBP and other transcription factors to coordinate promoter activity and gene expression through epigenetic and hormonal pathways [43,44,45,46,47,48,49]. Its role is conserved across diverse fruit species, including non-climacteric and monocot fruits [50,51].

Pectinesterase 1 (PE1) (LOC103721432) and Pectinase (EC 3.2.1.15) (LOC103706063) also exhibited missense variants with a moderate impact. These enzymes modify and degrade pectin, promoting cell wall softening. In tomato, peach, and strawberry, PE1 expression is tightly regulated during ripening and influenced by ethylene and auxin, correlating with the transition to softer textures [52,53,54,55,56,57,58]. Additionally, Galacturan (EC 3.2.1.67) (LOC103717066) carried a high-impact frameshift variant, suggesting further disruption of pectin metabolism.

#### 3.3.4. Implications for Breeding and Genetic Improvement

The comprehensive catalog of genomic variants identified in the Deglet Nour genome offers valuable insights for breeding programs aimed at improving date palm cultivars. The detection of moderate- and high-impact variants in key genes involved in sugar metabolism, fruit morphology, and firmness provides a rich source of candidate alleles that could be targeted to enhance fruit quality traits. For example, missense and frameshift mutations in sugar metabolism genes may influence fruit sweetness and maturation dynamics, traits highly valued in commercial cultivation. Similarly, variants in fruit shape and size regulators represent promising targets for modifying the fruit morphology and yield potential. Moreover, the identification of impactful mutations in firmness-related genes can inform selection for improved fruit texture and shelf life, critical attributes for marketability and consumer preference. The presence of a rich allele reservoir within Deglet Nour highlights its potential to contribute valuable genetic diversity by introducing novel traits into broader date palm breeding programs. However, it is important to note that the predicted effects of these alleles or allelic combinations on the phenotype are based on in silico analyses. Therefore, further functional genomics studies are necessary to validate their actual impact and to fully exploit this genetic variation in breeding efforts [59].

## 4. Materials and Methods

### 4.1. DNA Isolation and Sequencing

Leaf samples of the Deglet Nour variety were collected from Tozeur, southern Tunisia (Plot 38, Plan Jhim; 33°53′18.4″ N, 8°07′13.9″ E), and transported to the laboratory, where they were stored at −80 °C. DNA extraction was performed using the DNeasy Plant Mini Kit (Qiagen, Hilden, Germany), following the manufacturer’s protocol. The plant sample was identified as Deglet Nour based on the fruit morphological characteristics and confirmed through patented SSR marker analysis [60]. The DNA integrity was assessed by electrophoresis on a 1% agarose gel, while the DNA concentration was quantified using a Qubit 3.0 fluorometer (Thermo Fisher Scientific, Waltham, MA, USA). Library preparation and sequencing was performed at Macrogen Europe (Amsterdam, The Netherlands). Sequencing was carried out using the TruSeq DNA PCR-Free protocol on an Illumina NovaSeq platform (San Diego, CA, USA). The average library insert size was approximately 470 bp. A single, high-quality DNA extraction was used for the library preparation; negative extraction controls were not included given the rigorous clean laboratory conditions and the single-sample design of this study.

### 4.2. Pre-Processing of Raw Reads

Quality control of the raw reads (FASTQ format, 151 bp paired end, Phred +33) was performed by running FastQC (https://www.bioinformatics.babraham.ac.uk/projects/fastqc/ accessed on 14 July 2025). Illumina technical sequences and low-quality reads were removed using Trimmomatic version 0.40 [61] with the following parameters: LEADING = 20; TRAILING = 20; SLIDING WINDOW = 4:20. Reads shorter than 75 nucleotides were discarded. These parameters were selected to achieve high-quality read trimming while preserving enough data for accurate downstream genome assembly and variant calling.

### 4.3. Sequence Assembly and Annotation

The optimal k-mer size was estimated using KmerGenie v.1.7051 [62]. High-quality reads were assembled de novo with ABySS v2.3.10 [63] with the following parameters: k = 113 and b = 15 G. Scaffolding of the assembled contigs was performed with ntJoin v1.1.5 [64] with the parameters *w = 250*, *n = 2*, and *G = 1000*, employing the Barhee BC4 reference genome as a guide to enhance the contiguity and improve the ordering of sequence. Gaps were filled using Sealer v2.3.10 [65] with the following parameters: −*b 50G*, −*B 2000*, −*L 150*, −*P 10*, −*F 1000*, --*long-search*, and multiple k-mer sizes (83, 93, 103, 113, 123). Scaffolds with a minimum length of 1,000 nucleotides were retained. To further extend and merge the scaffolds, AlignGraph [66] was applied with the following parameters: *--distanceLow 150*, *--distanceHigh 1620*, −*kMer 113*, *--insertVariation 79*, *--coverage 10*, and *--fastMap*. Extended scaffolds underwent additional rounds of scaffolding and gap-filling using ntJoin and Sealer. Finally, genome annotation was performed using LiftOff v.1.6.3 [67], which applied a lift-over strategy to transfer gene models from the Barhee BC4 reference genome to the Deglet Nour assembly.

### 4.4. Assessment of Sequence Assembly

To evaluate the quality of the draft genome assembly, several metrics were calculated using the QUAST tool v.5.1 [68]. The assembly completeness was assessed by mapping the reads—excluding those initially aligned to the mitochondrial genome—to the final assembly using Bowtie v2.4.5 [69], with the —very-sensitive parameter.

The genome coverage was calculated by re-mapping the cleaned reads to the final assembly using Bowtie2 v2.4.5. The resulting alignment file (BAM) was processed using the *genomecov* function from bedtools v2.29.1 [70] to generate contiguous genomic intervals annotated with the per-base sequencing depth. Subsequently, the length-weighted mean sequencing depth was computed in R version 4.4.2 using the stats::weighted.mean() function, with each interval’s length used as the weighting factor.

The completeness of the final genome assembly was assessed using BUSCO [71] v5.8.3, with the “viridiplantae_odb12” lineage dataset.

Colinear blocks were identified using MCScanX v1.0.0 [72] with the following parameters: MATCH_SCORE = 50, GAP_PENALTY = −1, MATCH_SIZE = 5, E_VALUE = 1 × 10^−5^, UNIT_DIST = 10,000, and MAX_GAPS = 20. MCScanX analysis was performed using a BLASTP output [73] generated by comparing the Deglet Nour protein set to the Barhee BC4 reference proteome. BLASTP (v2.12.0) was run using the following parameters: -evalue 1 × 10^−10^ and -max_target_seqs 5. The resulting colinear blocks were visualized using SynVisio (https://synvisio.github.io accessed on 15 July 2025).

### 4.5. Identification of Transposable Elements

To identify transposable elements (TEs), we used RepeatMasker (v4.1.7-p1). Two Dfam_3.8 database partitions—partition 0 (“root”) and partition 5 (“Viridiplantae”)—as well as the final available version of Repbase (26 October 2018), were configured for use with RepeatMasker (v4.1.7-p1). DANTE (v0.1.9) [74] was also employed to achieve a more refined classification of the transposable elements into their respective classes, orders, and superfamilies. The outputs from RepeatMasker and DANTE were subsequently integrated into a unified dataset, allowing precise quantification and characterization of the transposable element content of the genome.

### 4.6. SNP Identification

High-quality reads were mapped to the Barhee BC4 reference genome (NCBI RefSeq assembly: GCF_009389715.1) using Bowtie v2.4.5 [69] with the --*very-sensitive-local* parameter. For SNP calling, a reference genome is required to provide a consistent framework for variant identification. The Barhee BC4 genome was selected for this purpose because it is currently the most complete and well-annotated date palm genome available.

The resulting BAM alignment file, along with the Barhee BC4 genome assembly and its annotation, was used as input for the RGAAT v1 tool [75]. To minimize the false positives in the raw SNP and INDEL calls generated by RGAAT, specific filtering thresholds were applied: the quality threshold for the reads was set to 30, the minimum read depth for the sequence variants was set to 3, the minimum allele depth was set to 3, and the minimum allele proportion required for calling a variant was set to 0.5.

### 4.7. Prediction of Variants Impacts on Coding Genes

SnpEff version 4.3 [76] was used to predict the potential effects of the identified SNPs and INDELs on the coding genes.

## 5. Conclusions

This study presents a draft genome assembly with moderate contiguity, along with the gene annotation, for the iconic Tunisian date palm cultivar Deglet Nour, offering valuable genomic insights into this economically and culturally significant species.

While some fragmentation remains, as reflected by a BUSCO completeness of 90.4%, the assembly benefits from a high mean sequencing depth of 94.26× and a scaffold N50 of 12.2 Kb, indicating strong contiguity for a genome assembled primarily from short-read Illumina data. Spanning 431 Mb, the assembly also shows robust overall read alignment, underscoring its accuracy and reliability. Together, these metrics confirm the assembly’s suitability for downstream genomic analyses. Comparative analysis with the Barhee BC4 reference genome revealed nearly 1.1 million SNPs and over 114,000 INDELs, including variants within the coding regions of genes associated with ripening, sugar metabolism, fruit firmness, and morphology.

These findings provide a foundation for fine-scale population genetic studies, trait-gene association analyses, GWAS validation, and marker-assisted selection. Functional annotations performed with SnpEff provide additional evidence that these variants play significant roles in critical developmental pathways, underscoring their potential impact on phenotypic traits and biological processes relevant to the species. Beyond its immediate applications, the Deglet Nour genome enables pan-genome construction and evolutionary studies across *Phoenix* species. It also lays the groundwork for downstream research in molecular breeding, conservation genetics, and the improvement of fruit traits. By providing a comprehensive catalog of variants, this work significantly advances the understanding of fruit maturation and quality in date palms, supporting efforts to improve cultivation practices and preserve the genetic heritage of this iconic cultivar.

## Figures and Tables

**Table 1:** Summary of the sequencing read quality and processing outcomes for the paired-end DNA libraries R1 and R2.

Sample ID	Raw Reads	Raw Read Q30 (%)	Paired Processed Reads	Processed Read Q30 (%)	Surviving Single Reads
DN_R1	172,691,117	93.95	157,872,866	90.40	9,766,454
DN_R2	172,691,117	94.81	157,872,866	94.81	2,324,394

**Table 2:** Comparative statistics of the Deglet Nour date palm assembly and previously published genomes assemblies.

Genomes	Size (Mb)	Number of Scaffolds	N50 (Kb)	Length of Sequences Anchored to LGs (Mb)
Al-Dous et al. [10] *	381	57,277	30.5	0
Al-Mssallem et al. [11] **	558	82,354	330.0	0
Hazzouri et al. [12] ***	772	2706	897.2	385.6
Present study	431	16,167	12.2	0

* GenBank reference number: GCA_000181215.2. ** GenBank reference number: GCA_000413155.1. *** GenBank reference number: GCA_009389715.1.

**Table 3:** Different types of variants identified in the Deglet Nour genome.

Variant Calling	Genome Deglet Nour
SNPs	1,062,681
Insertions	63,274
Deletions	51,033
Total	1,176,998

## Data Availability

The original data presented in the study are openly available in [NCBI] at [BioProject ID PRJNA1254475, BioSample SAMN48113132]. The genome assembly in FASTA format, genome annotation in GFF3 format, coding sequences (CDS) in FASTA format, and their corresponding protein translations are available in the Mendeley Data Repository under the following DOI: 10.17632/7kfrp6gzn6.1.

## Supplementary Materials

The following supporting information can be downloaded at https://www.mdpi.com/article/10.3390/ijms26146844/s1.

## Abbreviations

The following abbreviations are used in this manuscript:

bp	Base pair
CDS	Coding DNA sequence
Gbp	Giga base pair
GWAS	Genome-wide association study
INDELs	Insertions/deletions
Kb	Kilo base
KEGG	Kyoto Encyclopedia of Genes and Genomes
LINE	Long interspersed nuclear element
LTR	Long terminal repeat
Mb	Mega base
NCBI	National Center for Biotechnology Information
NGS	Next-generation sequencing
QTL	Quantitative trait loci
SINE	Short interspersed nuclear element
SNPs	Single nucleotide polymorphisms
TIR	Terminal inverted repeat
