# Supplementary material for: Comparative Genomics and Draft Genome Assembly of the Elite Tunisian Date Palm Cultivar Deglet Nour: Insights into the Genetic Variations Linked to Fruit Ripening and Quality Traits
Source: Int J Mol Sci. 2025 Jul 16;26(14):6844. doi: 10.3390/ijms26146844 (PMC12295771; doi:10.3390/ijms26146844)
Supplement: Supplementary file 1 [file ijms-26-06844-s001.zip › ijms-3674413-supplementary/Supplementary Figures.pdf]

Supplementary Figures for:

Comparative Genomics and Draft Genome Assembly of the Elite Tunisian Date Palm Cultivar Deglet Nour: Insights into the Genetic Variations Linked to Fruit Ripening and Quality Traits

Rahma Zarkouna [1], Afifa Hachef [1], Carmine Fruggiero [2], Gaetano Aufiero [2], Davide D'Angelo [2], Hedia Bourguiba [1], Maha Mezghani-Khemakhem [1], Nunzio D'Agostino [2,*] and Salwa Zehdi-Azouzi [1]

1 Laboratory of Molecular Genetics, Immunology and Biotechnology (LR99ES12), Faculty of Sciences of Tunis, University of Tunis El Manar, El Manar I, Tunis 2092, Tunisia; rahma.zarkouna@etudiant-fst.utm.tn (R.Z.); afifa.hachef@fst.utm.tn (A.H.); hedia.bourguiba@fst.utm.tn (H.B.); maha.mezghani@fst.utm.tn (M.M.); salwa.zehdi@fst.utm.tn (S.Z.)

2 Department of Agricultural Sciences, University of Naples Federico II, Piazza Carlo di Borbone 1, 80055 Portici, Italy; carmine.fruggiero@unina.it (C.F.); gaetano.aufiero@unina.it (G.A.); davide.dangelo@unina.it (D.D.)

* Correspondence: nunzio.dagostino@unina.it; Tel.: +39-0812539486

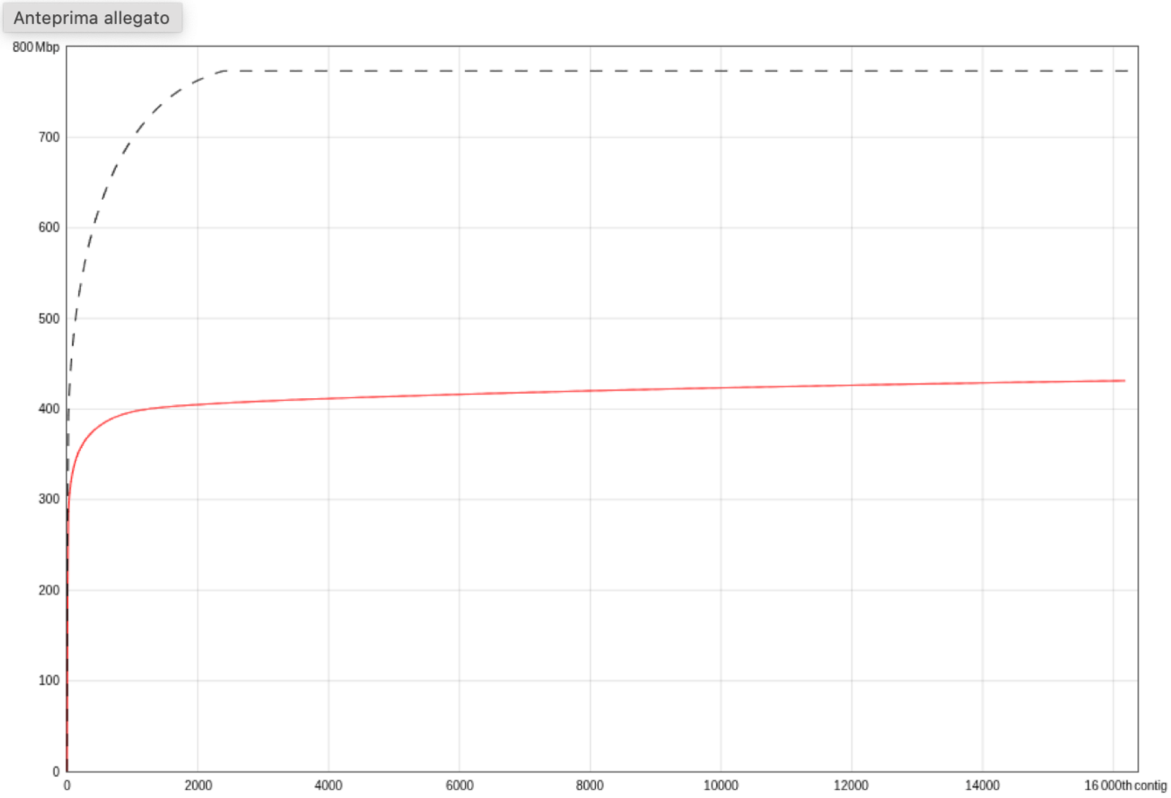

Figure S1. Cumulative length of the Deglet Nour genome (red line) compared to the reference genome Barhee BC4 (black dotted line) by QUAST.

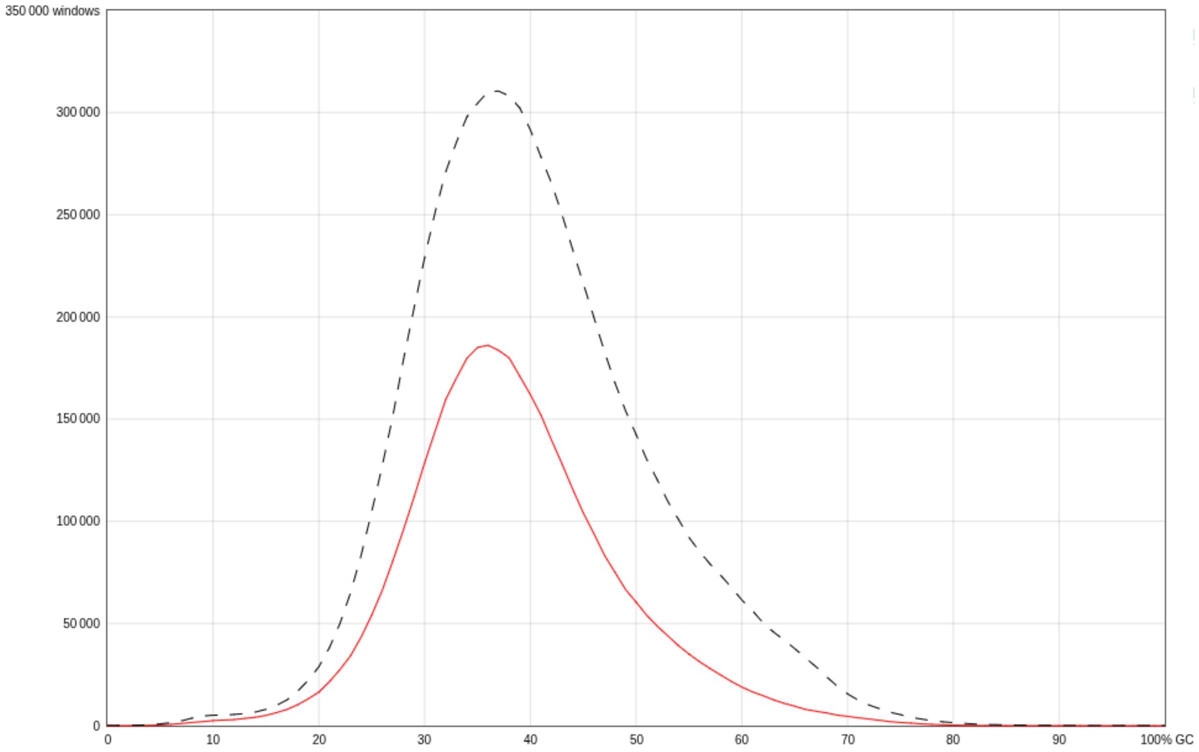

Figure S2. GC content of the Deglet Nour genome (red line) compared to reference genome Barhee BC4 (black dotted line).

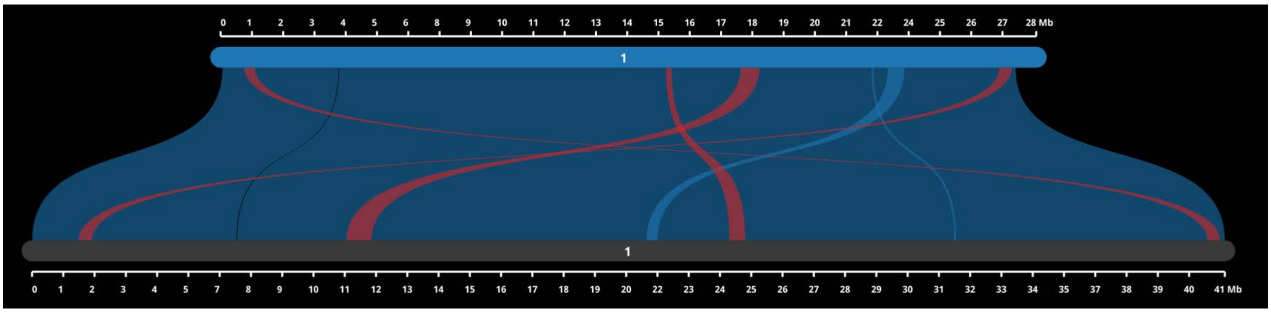

Figure S3. Pairwise alignment of the first scaffold of Deglet Nour (top) and chromosome 1 of the reference genome Barhee BC4 (bottom) using MCScanX. Collinear regions are shown in blue, while inverted regions are highlighted in red.
